# Three-Event Energy Detection with Adaptive Threshold for Spectrum Sensing in Cognitive Radio Systems

**DOI:** 10.3390/s20133614

**Published:** 2020-06-27

**Authors:** Alexandru Martian, Mahmood Jalal Ahmad Al Sammarraie, Călin Vlădeanu, Dimitrie C. Popescu

**Affiliations:** 1 Telecommunications Department, Polytechnic University of Bucharest, 061071 Bucharest, Romania; mahmood.alsammarraie@gmail.com (M.J.A.A.S.); calin.vladeanu@upb.ro (C.V.); 2 Department of Electrical and Computer Engineering, Old Dominion University, Norfolk, VA 23529, USA; dpopescu@odu.edu

**Keywords:** energy detection, spectrum sensing, cognitive radio, dynamic spectrum access, adaptive decision threshold

## Abstract

Implementation of dynamic spectrum access (DSA) in cognitive radio (CR) systems requires the unlicensed secondary users (SU) to implement spectrum sensing to monitor the activity of the licensed primary users (PU). Energy detection (ED) is one of the most widely used methods for spectrum sensing in CR systems, and in this paper we present a novel ED algorithm with an adaptive sensing threshold. The three-event ED (3EED) algorithm for spectrum sensing is considered for which an accurate approximation of the optimal decision threshold that minimizes the decision error probability (DEP) is found using Newton’s method with forced convergence in one iteration. The proposed algorithm is analyzed and illustrated with numerical results obtained from simulations that closely match the theoretical results and show that it outperforms the conventional ED (CED) algorithm for spectrum sensing.

## 1. Introduction

The expansion of wireless communication systems in all sectors of modern society over the past decade has prompted an increased demand in spectrum resources. In this context, the traditional static allocation of the radio frequencies leads to inefficient use of the spectrum [1,2], and cognitive radio (CR) systems have emerged as a meaningful alternative for improving the efficiency of spectrum usage through dynamic spectrum access (DSA) [3]. DSA allows the access of secondary users (SU) to licensed frequency bands when these are not actively used by licensed primary users (PU), and relies on spectrum sensing, which is performed by the SU to detect the presence of active PU.

Among the methods used for spectrum sensing, energy detection (ED) [4,5,6,7,8] is the most commonly used one, due to its simplicity as well as its almost universal applicability. Alternative spectrum sensing approaches use eigenvalue-based algorithms [9], covariance-based detection methods [10,11], cyclostationary feature detection [12,13], or compressive sensing [14,15]. A thorough and very recent survey on most known spectrum sensing methods can be found in [16].

An important drawback of the ED method is implied by its sensitivity to noise uncertainty [17], which has led to improved ED algorithms that outperform the classical energy detection (CED) method [4]. These include the modified ED method for spectrum sensing in [18], which uses the average value of the ED test statistic over multiple sensing events, as well as the three-event ED (3EED) algorithm in [19], which decides that a PU is active if the ED test statistic exceeds the sensing threshold in three consecutive sensing events that include the current sensing event along with the sensing events immediately before and after it. To improve the performance of ED in the presence of Laplacian noise, [20] proposes a spectrum sensing algorithm in which the square of the received signal amplitude in the ED test statistic is replaced by an exponent that ranges between 0 and 2. Furthermore, adaptive ED approaches have been established for dynamic scenarios, which adjust the duration of the sensing window based on the PU on/off activity [21] or change the sensing threshold in response to changes in the noise characteristics [22,23].

In this paper, we present a new adaptive ED algorithm for spectrum sensing, which is based on the 3EED algorithm [19], but in which the sensing threshold is adapted similar to [22], to optimize the decision error probability (DEP), which is a weighted sum of the probabilities of missed detection and false alarm [22,23]. We note that obtaining an accurate analytical expression for the optimal threshold is elusive as the expressions involved in finding the sensing threshold do not have closed-form expressions and require approximations. In order to underline the novelty of the current work, we will enumerate its contributions as compared to the previous similar and related works. In [22], the authors proposed a threshold adaptation method by minimizing the DEP under the Gaussian approximation, but for the simple CED algorithm. Hence, an exact expression of the threshold could be obtained directly as a solution of the optimization equation, using the properties of the *Q*-function. However, for more efficient algorithms than CED, regularly having more complex expressions for DEP, the optimization equation is not analytically solvable. Therefore, in this paper, we aim at extending the method from [22] to a more complex and efficient ED algorithm, such as 3EED [19]. Under the Gaussian approximation, the DEP for any ED algorithm can be written as an expression based on several *Q*-function terms. First, we have to prove that DEP is a convex function in the threshold value and then, we propose the use of a numerical method, such as Newton’s method, to iteratively determine the root of the analytically unsolvable optimization equation. However, the main drawback of iterative methods is the increased operating time, which is a critical issue for the spectrum sensing algorithms. In order to overcome this problem, we propose in this paper a transformation of the optimization function, such that the Newton’s method for the transformed function converges faster. By analyzing the monotonicity of the optimization function, we determine a transform that linearizes the function around the root. Thus, we manage to reduce the number of iterations to the minimum possible, i.e., one iteration. As a prior validation step for the current research, in [24], we tested only empirically the performance of the adaptive threshold 3EED algorithm, where we derived the threshold value by using a brute-force search. Finally, in the current paper, we derive an analytical approximate expression of the adaptive threshold for 3EED by using a fast convergence numerical method. Moreover, we consider that this method can be generalized for most if not all ED-based spectrum sensing algorithms.

The rest of the paper is structured as follows: in Section 2, we briefly present the CED and 3EED algorithms for spectrum sensing, followed by a discussion on optimizing the sensing threshold in Section 3. The proposed 3EED algorithm with an adaptive threshold is formally stated in Section 4. In Section 5, we illustrate the performance of the proposed algorithm with numerical results obtained from simulation and compare it to that of the adaptive CED in [22], and we conclude the paper with final remarks in Section 6.

## 2. Spectrum Sensing by Three-Event Energy Detection (3EED)

Let y(n) be the signal at the SU receiver, which consists of an active PU signal with average power $\sigma_{s}^{2}$ corrupted by additive white Gaussian noise (AWGN) variable with variance $\sigma_{n}^{2}$ when the channel is busy, or just of the AWGN when the channel is idle (PU is not active). We denote by $E_{i}$ the value of the received signal energy estimated during the *i*-th sensing slot that consists of *N* samples, λ the ED sensing threshold, and $q_{i}$ the binary decision variable {0,1} for the *i*-th spectrum sensing slot. With these notations, in CED correct detection of the PU signal (active/inactive) implies setting $q_{i}=1$, that is, the channel is “busy”, if $E_{i}>\lambda$ when y(n) is implied by an active PU signal corrupted by noise, and $q_{i}=0$, that is, the channel is “idle”, if $E_{i}\leq \lambda$ when y(n) corresponds to a noise only term. We note that, when the number of samples *N* is sufficiently large, the tail region of the probability density function of the received signal energy $E_{i}$ for values above the sensing threshold is approximated by a normal distribution [25] such that the standard *Q*-function [26] can be used to approximate the probabilities of detection and false alarm for CED as [18]

(1) $$P_{fa}^{CED}\left(\lambda ,\sigma_{n}^{2}\right)=\mathrm{Prob}\left[E_{i}>\lambda |\mathrm{channel}\;\mathrm{is}\;“\mathrm{idle}”\right]=Q\left(\frac{\frac{\lambda }{N}-\sigma_{n}^{2}}{\sigma_{n}^{2}\sqrt{\frac{2}{N}}}\right)$$

and

(2) $$P_{d}^{CED}\left(\lambda ,\sigma_{s}^{2},\sigma_{n}^{2}\right)=\mathrm{Prob}\left[E_{i}>\lambda |\mathrm{channel}\;\mathrm{is}\;“\mathrm{busy}”\right]=Q\left[\frac{\frac{\lambda }{N}-\sigma_{s}^{2}-\sigma_{n}^{2}}{\left(\sigma_{s}^{2}+\sigma_{n}^{2}\right)\sqrt{\frac{2}{N}}}\right].$$

As the name suggests, the 3EED algorithm [19] relies on ED in up to three consecutive sensing slots to decide if the signal y(n) at the SU receiver is implied by an active PU signal or not. Specifically, similar to CED, if the energy in the current spectrum sensing slot *i* exceeds the sensing threshold, $E_{i}>\lambda$, then the 3EED algorithm decides that a PU is active and sets $q_{i}=1$. However, if $E_{i}\leq \lambda$, the algorithm checks the energy level estimated in the previous slot, $E_{i-1}$, to decide that a PU signal is active and set $q_{i}=1$ if $E_{i-1}>\lambda$, or to move on to the next time slot i+1 to estimate $E_{i+1}$ and make the final decision that PU signal is active setting $q_{i}=1$ if $E_{i+1}>\lambda$, or inactive setting $q_{i}=0$ if $E_{i+1}\leq \lambda$. Thus, if the PU signal is identified as active in *any* of the three consecutive sensing slots, *i*, i−1, and i+1, the algorithm returns $q_{i}=1$ corresponding to a “busy” channel, while if the PU signal is determined to be inactive in *all* three consecutive time slots the algorithm returns $q_{i}=0$ corresponding to an “idle” channel. The formal statement of the 3EED algorithm is given in Algorithm 1.
**Algorithm 1** The 3EED Algorithm for Spectrum SensingInput: sensing threshold λOutput: output result1:**for** each spectrum sensing slot *i* **do**2:    Estimate received signal energy $E_{i}$ and save its value3:    **if** $E_{i}>\lambda$ **then**4:        Set $q_{i}=1$⟶ PU active/Channel “busy”5:    **else**6:        Read $E_{i-1}$ (saved in slot *i*-1)7:        **if** $E_{i-1}>\lambda$ **then**8:           Set $q_{i}=1$⟶ PU active/Channel “busy”9:        **else**10:           Estimate $E_{i+1}$11:           **if** $E_{i+1}>\lambda$ **then**12:               Set $q_{i}=1$⟶ PU active/Channel “busy”13:           **else**14:               Set $q_{i}=0$⟶ PU not active/Channel “idle”15:**return**$q_{i}$

The probabilities of false alarm and detection for the 3EED algorithm for spectrum sensing are expressed in terms of the probabilities of false alarm and detection for the CED algorithm, $P_{fa}^{CED}$ and $P_{d}^{CED}$, respectively as [19]:

(3) $$\begin{array}{ccc} P_{fa}^{3EED} & = & P_{fa}^{CED}+\left(1-P_{fa}^{CED}\right)\cdot \\ & & \;\left(\frac{T-B-1}{T-B}P_{fa}^{CED}+\frac{1}{T-B}P_{d}^{CED}\right)\cdot \\ & & \;\left[1+\frac{T-B-1}{T-B}\left(1-P_{fa}^{CED}\right)+\frac{1}{T-B}\left(1-P_{d}^{CED}\right)\right] \end{array}$$

and

(4) $$\begin{array}{ccc} P_{d}^{3EED} & = & P_{d}^{CED}+\left(1-P_{d}^{CED}\right)\cdot \\ & & \;\left(\frac{1}{B}P_{fa}^{CED}+\frac{B-1}{B}P_{d}^{CED}\right)\cdot \\ & & \;\left[1+\frac{1}{B}\left(1-P_{fa}^{CED}\right)+\frac{B-1}{B}\left(1-P_{d}^{CED}\right)\right], \end{array}$$

where *T* represents the total duration of the transmission cycle that consists of *B* consecutive slots during which the channel is busy followed by a number of T−B slots in which the channel is idle. We note that the ratio α=B/T represents the spectrum utilization ratio of the PU (that is the fraction of time the channel is actively used by the PU).

The decision threshold λ is determined based on a desired performance level that is specified in terms of a target probability of false alarm value, $P_{fa}$, as it is usually the case with constant false alarm rate (CFAR) detectors [19],

(5) $$\lambda =\left[Q^{-1}\left(1+\sqrt[{3}]{P_{fa}-1}\right)\sqrt{2N}+N\right]\sigma_{n}^{2}.$$

We note that the threshold must be adapted when changes in the noise variance occur, in order to keep the probability of false alarm at the desired value. We also note that, by considering only the probability of false alarm in setting the sensing threshold, the SU is favored, since by setting the value of $P_{fa}$ low the SU has more chances of utilizing the spectrum. However, in the case of a mis-detection, when the PU is active but the channel is incorrectly detected as “idle”, the SU transmission will interfere with the active PU signal and will negatively affect the PU performance. Thus, in order for spectrum sensing to be beneficial to both PU and SU, threshold setting should consider both the probability of false alarm and the probability of mis-detection.

## 3. Optimizing the 3EED Sensing Threshold to Minimize the Decision Error Probability

To optimize the decision threshold of the 3EED algorithm for spectrum sensing we use the DEP as the performance metric, which is formally defined as [22]:

(6) $$P_{e}\left(\lambda ,\alpha ,\sigma_{s}^{2},\sigma_{n}^{2}\right)=\left(1-\alpha \right)P_{fa}^{3EED}+\alpha \left(1-P_{d}^{3EED}\right).$$

Analyzing expression (6) we note that the DEP is a function of the sensing threshold λ, the PU spectrum utilization ratio α, the average power of the PU signal $\sigma_{s}^{2}$, and the noise variance $\sigma_{n}^{2}$. Furthermore, both terms in the DEP expression (6) are related to decision errors in the spectrum sensing process:

- The first term in (6) is implied by the probability of false alarm and corresponds to the case when a given sensing slot is found busy without an active PU transmission in this slot. False alarms occur only when the channel is idle and the term is weighted by the 1−α factor corresponding to the fraction of time when the PU is not active.
- The second term in (6) corresponds to the probability of mis-detection and is associated with the case when a given sensing slot is found idle while an active PU transmission is actually in progress in this slot. Mis-detections occur when the channel is busy and the term is weighted by the α factor corresponding to the fraction of time when the PU is active.

Thus, the DEP provides a trade-off between the false alarm and mis-detection performance, and allows optimization of the decision threshold for the 3EED algorithm by setting it to minimize the DEP (6), that is

(7) $$\lambda_{opt}=\arg\min_{\lambda }\;P_{e}\left(\lambda ,\alpha ,\sigma_{s}^{2},\sigma_{n}^{2}\right).$$

Introducing (3) and (4) in (6), we rewrite the expression of DEP as

(8) $$\begin{array}{ccc} P_{e}\left(\lambda ,\alpha ,\sigma_{s}^{2},\sigma_{n}^{2}\right)\; & = & \;\left(1-\alpha \right)\left\{1+{\left[P_{fa}^{CED}\left(\lambda ,\sigma_{n}^{2}\right)-1\right]}^{3}\right\}\\ & + & \;\alpha {\left[1-P_{d}^{CED}\left(\lambda ,\sigma_{s}^{2},\sigma_{n}^{2}\right)\right]}^{3}, \end{array}$$

For the sake of simplicity, let us introduce two variables denoted as $a(\lambda ,\sigma_{n}^{2})$ and $b(\lambda ,\sigma_{s}^{2},\sigma_{n}^{2})$, which are the arguments of the *Q*-function in (1) and (2), respectively, and expressed as

(9) $$a\left(\lambda ,\sigma_{n}^{2}\right)=\frac{\lambda }{\sqrt{2N}\sigma_{n}^{2}}-\sqrt{\frac{N}{2}}$$

and

(10) $$b\left(\lambda ,\sigma_{s}^{2},\sigma_{n}^{2}\right)=\frac{\lambda }{\sqrt{2N}\left(\sigma_{s}^{2}+\sigma_{n}^{2}\right)}-\sqrt{\frac{N}{2}}.$$

Using the expressions (9) and (10), we can rewrite the DEP function from (8) as following:

(11) $$\begin{array}{ccc} P_{e}\left(\lambda ,\alpha ,\sigma_{s}^{2},\sigma_{n}^{2}\right)\; & = & \;\left(1-\alpha \right)\left\{1+{\left[Q\left(a\right)-1\right]}^{3}\right\}+\alpha {\left[1-Q\left(b\right)\right]}^{3}, \end{array}$$

We note that finding an accurate closed-form expression for $\lambda_{opt}$ is elusive as the expression of the DEP metric to be optimized has already been approximated when expressions of $P_{fa}^{3EED}$ and $P_{d}^{3EED}$ in terms of the *Q*-functions have been used to obtain (11), and resorting to further approximations of the *Q*-function in terms of simpler elementary functions will affect the accuracy of the optimal threshold value obtained. Thus, we focus on numerical optimization of the DEP metric (11), which takes advantage of its convexity properties.

Specifically, we notice that the DEP function has four variables, i.e., λ, α, $\sigma_{s}^{2}$, and $\sigma_{n}^{2}$. However, the optimization aims only at the threshold variable, λ. Therefore, the other three variables are assumed to be constant for this optimization. In order to perform the threshold optimization, the DEP function from (6) and (11) has to be a convex function in λ. Therefore, we provide the next theorem.

**Theorem** **1.** *The DEP function given by (11) is a convex function in λ.*

**Proof.**  Let us introduce the following notations for the functional terms from (11):

(12) $$\begin{array}{ccc} T_{1}\left(a\right)\; & = & \;1+{\left[Q\left(a\right)-1\right]}^{3}\\ T_{2}\left(b\right)\; & = & \;{\left[1-Q\left(b\right)\right]}^{3} \end{array}$$

Analyzing the second derivative of both functions from (12) with respect to *a* and *b*, respectively, one can notice that both have a unique and identical inflection point (i.e., the solution of $T_{1}^{\prime \prime }\left(a\right)=0$ and $T_{2}^{\prime \prime }\left(b\right)=0$). The value of this common inflection point is the solution of the following transcendental equation for x=a or x=b:

(13) $$\begin{array}{ccc} 2Q^{\prime }\left(x\right)\; & = & \;x\left[Q\left(x\right)-1\right] \end{array}$$

An approximate value for the solution of the Equation (13) can be obtained using numerical methods or graphically, i.e., $x_{0}=0.76527\ldots$. Considering this approximate value of $x_{0}$ and using the relations in (9) and (10), we can determine the values of the inflection point values of $\lambda_{inflec}^{\left(1\right)}$ and $\lambda_{inflec}^{\left(2\right)}$ for the functions $T_{1}\left(a\right)$ and $T_{2}\left(b\right)$, respectively, from (12):

(14) $$\begin{array}{ccc} \lambda_{inflec}^{\left(1\right)}\; & = & \;\sqrt{2N}\sigma_{n}^{2}\left(x_{0}+\sqrt{\frac{N}{2}}\right)\\ \lambda_{inflec}^{\left(2\right)}\; & = & \;\sqrt{2N}\left(\sigma_{s}^{2}+\sigma_{n}^{2}\right)\left(x_{0}+\sqrt{\frac{N}{2}}\right) \end{array}$$

In order to evaluate the convexity of the functions from (12), we have to estimate the values in the inflection points, given by (14), of the first derivative of these functions with respect to λ, respectively, denoted as $T_{1}^{\prime }\left(\lambda =\lambda_{inflec}^{\left(1\right)}\right)$ and $T_{2}^{\prime }\left(\lambda =\lambda_{inflec}^{\left(2\right)}\right)$. Analyzing these first derivative functions, we noticed that $T_{1}^{\prime }\left(\lambda_{inflec}^{\left(1\right)}\right)<0$ and $T_{2}^{\prime }\left(\lambda_{inflec}^{\left(2\right)}\right)>0$. Therefore, it results that $T_{1}\left(\lambda \right)$ is convex in λ for $\lambda \geq \lambda_{inflec}^{\left(1\right)}$ and $T_{2}\left(\lambda \right)$ is convex in λ for $\lambda \leq \lambda_{inflec}^{\left(2\right)}$.

Also, we note that the DEP expression from (11), with the notations from (12), is a linear combination of the two convex functions $T_{1}\left(\lambda \right)$ and $T_{2}\left(\lambda \right)$ for the intersection set $\lambda_{inflec}^{\left(1\right)}\leq \lambda \leq \lambda_{inflec}^{\left(2\right)}$:

(15) $$\begin{array}{ccc} P_{e}\left(\lambda ,\alpha ,\sigma_{s}^{2},\sigma_{n}^{2}\right)\; & = & \;\left(1-\alpha \right)T_{1}\left(\lambda ,\alpha ,\sigma_{n}^{2}\right)+\alpha T_{2}\left(\lambda ,\alpha ,\sigma_{s}^{2},\sigma_{n}^{2}\right), \end{array}$$

We also know that the spectrum utilization ratio of the PU is a positive number, i.e., $\alpha \in \left[0,1\right]$ and therefore, both coefficients of the linear expression from (15) are positive numbers.

Finally, Theorem 2.10 from page 52 in [27] states that a linear combination of two convex functions on a set, with non-negative coefficients, is also a convex function. Hence, we can conclude that the DEP expression from (11) is a convex function in λ on the set $\lambda_{inflec}^{\left(1\right)}\leq \lambda \leq \lambda_{inflec}^{\left(2\right)}$. □

Now, because the DEP function is convex as stated by Theorem 1, we can determine the optimum threshold from (7) by solving the following equation to find the critical points of the DEP (6):

(16) $$\frac{\partial P_{e}\left(\lambda ,\alpha ,\sigma_{s}^{2},\sigma_{n}^{2}\right)}{\partial \lambda }=0$$

Next, using the DEP expression from (11) we obtain

(17) $$\begin{array}{ccc} \frac{\partial P_{e}}{\partial \lambda } & = & 3\left(1-\alpha \right){\left[Q(a)-1\right]}^{2}\frac{dQ(a)}{da}\frac{\partial a}{\partial \lambda }\\ & - & 3\alpha {\left[Q(b)-1\right]}^{2}\frac{dQ(b)}{db}\frac{\partial b}{\partial \lambda } \end{array}$$

where $a(\lambda ,\sigma_{n}^{2})$ and $b(\lambda ,\sigma_{s}^{2},\sigma_{n}^{2})$ were previously introduced in (9) and (10).

Using the expression of the first derivative of the *Q*-function

(18) $$\frac{dQ(x)}{dx}=-\frac{1}{\sqrt{2\pi }}e^{-x^{2}/2}$$

the threshold optimization Equation (16) becomes

(19) $$\frac{Q(a)-1}{Q(b)-1}=A\left(\alpha ,\gamma \right)e^{-(b^{2}-a^{2})/4}$$

where $\gamma =\sigma_{s}^{2}/\sigma_{n}^{2}$ is the signal-to-noise ratio (SNR) and

(20) $$A\left(\alpha ,\gamma \right)=\sqrt{\frac{\alpha }{1-\alpha }}\cdot \sqrt{\frac{1}{1+\gamma }}.$$

We note that Equation (19) is transcendental, which makes it difficult to find a closed-form solution for the optimal threshold $\lambda_{opt}$, and the alternative is to obtain a numerical solution for it as discussed in the following section.

## 4. Numerical Approach to Finding the Optimal 3EED Sensing Threshold

The numerical approach used to find $\lambda_{opt}$, the optimal threshold for the 3EED spectrum sensing, is based on the application of Newton’s iterative method [28] for Equation (19) rewritten as

(21) $$\underset{f(\lambda )}{\underset{︸}{\frac{Q(a)-1}{Q(b)-1}-A\left(\alpha ,\gamma \right)e^{-(b^{2}-a^{2})/4}}}=0\;.$$

As the rate of convergence for Newton’s method is quadratic, with proper initialization, a solution to (21) that approximates well the optimal sensing threshold $\lambda_{opt}$ can be found in only a few iterations of the type [28]

(22) $$\lambda_{n+1}=\lambda_{n}-\frac{f\left(\lambda_{n}\right)}{f^{\prime }\left(\lambda_{n}\right)}.$$

Furthermore, the proposed approach uses the *Q*-function approximation [29]

(23) $$Q\left(x\right)≅\frac{\left(1-e^{-1.4x}\right)e^{-x^{2}/2}}{1.135\sqrt{2\pi }x},\;\;\;\;x\geq 0$$

to rewrite the Q(a) and Q(b) terms in (21).

We have to note that the initialization of Newton’s iterative method, used to solve the optimization equation from (21), and its convergence are strongly related to the convexity of the DEP function. According to the DEP function’s convexity demonstrated in Theorem 1, we know that the best initialization values of λ should be the values of the inflection points, i.e., either $\lambda_{inflec}^{\left(1\right)}$ or $\lambda_{inflec}^{\left(2\right)}$ given in (14). However, for the analytical derivation of the optimum value of λ, the use of $\lambda_{inflec}^{\left(1\right)}$ or $\lambda_{inflec}^{\left(2\right)}$ in the expressions of the optimization function from (21) and its derivative function would have complex expressions. Instead, we prefer to use the values of λ for which the argument variables *a* and *b* in the optimization Equation (21) take null values, i.e., a=0 or b=0. Hence, all terms multiplied by *a* or *b* will be eliminated from the final expressions. The λ values that are the solutions for the equations $a(\lambda ,\sigma_{n}^{2})=0$ and $b(\lambda ,\sigma_{s}^{2},\sigma_{n}^{2})=0$, denoted as $\lambda_{a,0}$ and $\lambda_{b,0}$, respectively, result from (9) and (10) as

(24) $$\lambda_{a,0}=N\sigma_{n}^{2}$$

and

(25) $$\lambda_{b,0}=N\sigma_{n}^{2}\left(1+\gamma \right)$$

It is important to note that the difference between the values of $\lambda_{a,0}$ and $\lambda_{b,0}$ and the values of $\lambda_{inflec}^{\left(1\right)}$ and $\lambda_{inflec}^{\left(2\right)}$ is almost neglectable. Using the expressions from (14), (24) and (25), it can be easily demonstrated that the relative difference between these values is given by:

(26) $$\begin{array}{c} \frac{\lambda_{inflec}^{\left(2\right)}-\lambda_{b,0}}{\lambda_{inflec}^{\left(2\right)}}=\frac{\lambda_{inflec}^{\left(1\right)}-\lambda_{a,0}}{\lambda_{inflec}^{\left(1\right)}}=\frac{x_{0}}{x_{0}+\sqrt{\frac{N}{2}}} \end{array}$$

Considering that $x_{0}=0.76527\ldots$ and *N* takes values of the order of tens of thousands samples, then it results in a value for the relative difference in (26) that is less than 1%.

Hence, considering all the above, we will use the values of $\lambda_{a,0}$ and $\lambda_{b,0}$ as initial values for Newton’s method.

Since (23) is valid only for positive arguments of the *Q*-function and the arguments $a(\lambda ,\sigma_{n}^{2})$ and $b(\lambda ,\sigma_{s}^{2},\sigma_{n}^{2})$ in (21) can take both positive and negative values, as can be observed from (9) and (10), the approximation is applied in conjunction with the symmetry property of the *Q*-function, Q(−x)=1−Q(x). Specifically, the following three distinct cases will be analyzed separately to provide a numerical solution for Equation (21): (i)b<a≤0, (ii)a>0,b≤0, and (iii)a>b≥0.

We have to note that the domain of λ values is separated into these three sub-domains ((i), (ii), and (iii)), where the value of $\lambda_{a,0}$ delimits cases (i) and (ii) and $\lambda_{b,0}$ delimits cases (ii) and (iii). Therefore, $\lambda_{a,0}$ will be used as the initial value for the iterative method in case (i) and $\lambda_{b,0}$ will be used as the initial value for the iterative method in case (iii). Instead, for case (ii), we can use either $\lambda_{a,0}$ or $\lambda_{b,0}$ as the initial value, because the minimum DEP can be reached from either side of the interval with a similar accuracy.

### 4.1. Case (i)b<a≤0

Using the symmetry property of the *Q*-function, Equation (21) can be rewritten as:

(27) $$f\left(\lambda \right)=\frac{Q(-a)}{Q(-b)}-A\left(\alpha ,\gamma \right)e^{-(b^{2}-a^{2})/4}=0,$$

which, upon using the approximation (23) becomes:

(28) $$\underset{f_{i}\left(\lambda \right)}{\underset{︸}{\frac{1-e^{1.4a}}{a}\frac{b}{1-e^{1.4b}}-A\left(\alpha ,\gamma \right)e^{-3\left(b^{2}-a^{2}\right)/4}}}=0$$

Denoting the function in the left-hand side of (28) by $f_{i}\left(\lambda \right)$, and initializing Newton’s iteration with the value $\lambda_{a,0}$ from (24) we obtain

(29) $$f_{i}\left(\lambda_{a,0}\right)=\frac{1.4b\left(\lambda_{a,0}\right)}{e^{1.4b\left(\lambda_{a,0}\right)}-1}-A\left(\alpha ,\gamma \right)e^{-0.75b^{2}\left(\lambda_{a,0}\right)}$$

(30) $$\begin{array}{ccc} f_{i}^{\prime }\left(\lambda_{a,0}\right) & = & \frac{0.98}{\sqrt{2N}\sigma_{n}^{2}}\frac{b\left(\lambda_{a,0}\right)}{e^{1.4b\left(\lambda_{a,0}\right)}-1}-\frac{1.4}{\sqrt{2N}\sigma_{n}^{2}\left(1+\gamma \right)}\cdot \left[\frac{1-e^{1.4b\left(\lambda_{a,0}\right)}+1.4b\left(\lambda_{a,0}\right)e^{1.4b\left(\lambda_{a,0}\right)}}{{\left(e^{1.4b\left(\lambda_{a,0}\right)}-1\right)}^{2}}\right]\\ & + & \frac{3A(\alpha ,\gamma )}{2}e^{-3b^{2}\left(\lambda_{a,0}\right)/4}b\left(\lambda_{a,0}\right)\frac{1}{\sqrt{2N}\sigma_{n}^{2}\left(1+\gamma \right)} \end{array}$$

The derivative $f_{i}^{\prime }\left(\lambda_{a,0}\right)$ is given by the expression (30), which, together with (29), can be used to start Newton’s iterations (22) to obtain a numerical solution for the optimal sensing threshold $\lambda_{opt}^{\left(i\right)}$.

### 4.2. Case (ii)a>0,b≤0

Following a similar approach as in the previous case but in which the symmetry property of the *Q*-function is used only for the Q(b) term, Equation (21) is rewritten in this case as:

(31) $$f\left(\lambda \right)=\frac{1-Q(a)}{Q(-b)}-A\left(\alpha ,\gamma \right)e^{-(b^{2}-a^{2})/4}=0,$$

for which approximation (23) implies that

(32) $$\begin{array}{c} \overset{f_{ii}\left(\lambda \right)}{\overset{︷}{\frac{b}{e^{1.4b}-1}\cdot \left[1.135\sqrt{2\pi }-\frac{\left(1-e^{-1.4a}\right)e^{-a^{2}/2}}{a}\right]-A\left(\alpha ,\gamma \right)e^{-(3b^{2}-a^{2})/4}}}=0 \end{array}$$

Denoting the function in the left-hand side of (32) by $f_{ii}\left(\lambda \right)$, and initializing Newton’s iteration with the value $\lambda_{b,0}$ from (25) we obtain

(33) $$\begin{array}{ccc} f_{ii}\left(\lambda_{b,0}\right)\; & = & \;\frac{1.135\sqrt{2\pi }}{1.4}-\left[1-e^{-1.4a(\lambda_{b,0})}\right]\frac{e^{-0.5a^{2}\left(\lambda_{b,0}\right)}}{1.4a(\lambda_{b,0})}-A\left(\alpha ,\gamma \right)e^{0.25a^{2}\left(\lambda_{b,0}\right)} \end{array}$$

The derivative $f_{ii}^{\prime }\left(\lambda_{b,0}\right)$ is given by expression (34), which, together with (33), can now be used to start Newton’s iterations (22) to obtain a numerical solution for the optimal sensing threshold $\lambda_{opt}^{\left(ii\right)}$.

(34) $$\begin{array}{ccc} f_{ii}^{\prime }\left(\lambda_{b,0}\right) & = & -\frac{1}{2\sqrt{2N}\sigma_{n}^{2}\left(1+\gamma \right)}\cdot \left[1.135\sqrt{2\pi }-\frac{\left(1-e^{-1.4a\left(\lambda_{b,0}\right)}\right)e^{-\frac{a^{2}\left(\lambda_{b,0}\right)}{2}}}{a\left(\lambda_{b,0}\right)}\right]\\ & + & \frac{\left(1-a^{2}\left(\lambda_{b,0}\right)\right)\left(1-e^{-1.4a\left(\lambda_{b,0}\right)}\right)e^{-\frac{a^{2}\left(\lambda_{b,0}\right)}{2}}}{1.4\sqrt{2N}\sigma_{n}^{2}a^{2}\left(\lambda_{b,0}\right)}-\frac{e^{-1.4a\left(\lambda_{b,0}\right)}e^{-\frac{a^{2}\left(\lambda_{b,0}\right)}{2}}}{\sqrt{2N}\sigma_{n}^{2}a\left(\lambda_{b,0}\right)}\\ & - & \frac{A\left(\alpha ,\gamma \right)a\left(\lambda_{b,0}\right)}{2\sqrt{2N}\sigma_{n}^{2}}e^{\frac{a^{2}\left(\lambda_{b,0}\right)}{4}} \end{array}$$

In case (ii), we have used $\lambda_{b,0}$ as the initial value, but we can obtain similar expressions for $f_{ii}\left(\lambda \right)$ and $f_{ii}^{\prime }\left(\lambda \right)$ with $\lambda_{a,0}$ as the initial value, as in (33) and (34), respectively, with similar performance results for Newton’s method.

### 4.3. Case (iii)a>b≥0

In this case, the approximation from (23) can be used directly in (21) to rewrite it as expression (35). Denoting the function in the left-hand side of (35) by $f_{iii}\left(\lambda \right)$ and initializing it in this case with the same value $\lambda_{b,0}$ in (25) we obtain the expressions for $f_{iii}\left(\lambda_{b,0}\right)$ and $f_{iii}^{\prime }\left(\lambda_{b,0}\right)$ as shown in (36) and (37), respectively, which can be used to start Newton’s iterations (22) to obtain a numerical solution for the optimal sensing threshold $\lambda_{opt}^{\left(iii\right)}$.

(35) $$\underset{f_{iii}\left(\lambda \right)}{\underset{︸}{\left[1.135\sqrt{2\pi }-\frac{\left(1-e^{-1.4a}\right)e^{-a^{2}/2}}{a}\right]\cdot \frac{b}{1.135\sqrt{2\pi }b-\left(1-e^{-1.4b}\right)e^{-b^{2}/2}}-A\left(\alpha ,\gamma \right)e^{-(b^{2}-a^{2})/4}}}=0$$

(36) $$f_{iii}\left(\lambda_{b,0}\right)=\frac{1}{1.135\sqrt{2\pi }-1.4}\cdot \left[1.135\sqrt{2\pi }-\frac{\left(1-e^{-1.4a\left(\lambda_{b,0}\right)}\right)e^{-a^{2}\left(\lambda_{b,0}\right)/2}}{a\left(\lambda_{b,0}\right)}\right]-A\left(\alpha ,\gamma \right)e^{a^{2}\left(\lambda_{b,0}\right)/4}$$

(37) $$\begin{array}{ccc} f_{iii}^{\prime }\left(\lambda_{b,0}\right) & = & \frac{e^{-a^{2}\left(\lambda_{b,0}\right)/2}}{\left(1.135\sqrt{2\pi }-1.4\right)\sqrt{2N}\sigma_{n}^{2}a\left(\lambda_{b,0}\right)}\cdot \left\{\frac{\left[1-e^{-1.4a\left(\lambda_{b,0}\right)}\right]\left[a^{2}\left(\lambda_{b,0}\right)+1\right]}{a\left(\lambda_{b,0}\right)}-1.4e^{-1.4a\left(\lambda_{b,0}\right)}\right\}\\ & - & \frac{1.96}{2{\left(1.135\sqrt{2\pi }-1.4\right)}^{2}\sqrt{2N}\sigma_{n}^{2}\left(1+\gamma \right)}\cdot \left[1.135\sqrt{2\pi }-\frac{\left(1-e^{-1.4a\left(\lambda_{b,0}\right)}\right)e^{-a^{2}\left(\lambda_{b,0}\right)/2}}{a\left(\lambda_{b,0}\right)}\right]\\ & - & \frac{A\left(\alpha ,\gamma \right)a\left(\lambda_{b,0}\right)e^{a^{2}\left(\lambda_{b,0}\right)/4}}{2\sqrt{2N}\sigma_{n}^{2}} \end{array}$$

### 4.4. One-Step Numerical Solution and the 3EED Algorithm with Adaptive Threshold

The rate of convergence for Newton’s iterations and the number of steps *n* needed to find a numerical solution $\lambda_{opt}^{\left(j\right)}$ to the optimal sensing threshold for each of the three cases outlined in the previous sections depend on the monotonicity of the corresponding $f_{j}\left(\lambda \right)$ function,

(38) $$\lambda_{opt}^{\left(j\right)}≅\lambda_{n+1}=\lambda_{n}-\frac{f_{j}\left(\lambda_{n}\right)}{f_{j}^{\prime }\left(\lambda_{n}\right)},\;j\in \left\{i,ii,iii\right\},$$

as well as on the initialization

(39) $$\lambda_{0}^{\left(j\right)}=\left\{\begin{array}{cc} \lambda_{a,0} & \mathrm{for}\;\mathrm{case}\;\;j=i\\ \lambda_{b,0} & \mathrm{for}\;\mathrm{cases}\;j=ii,iii \end{array}\right.$$

In order to improve convergence and minimize the overhead associated with finding the optimal sensing threshold, we propose to use the alternative function

(40) $$F_{j}\left(\lambda \right)=\ln\left[1+|f_{j}\left(\lambda \right)|\right]$$

having the derivative expressed as

(41) $$F_{j}^{\prime }\left(\lambda \right)=\frac{f_{j}\left(\lambda \right)\cdot f_{j}^{\prime }\left(\lambda \right)}{|f_{j}\left(\lambda \right)|+f_{j}^{2}\left(\lambda \right)},$$

which yields a good approximation for the optimal sensing threshold in only one iteration

(42) $$\lambda_{opt}^{\left(j\right)}≅\lambda_{1}=\lambda_{0}^{\left(j\right)}-\frac{F_{j}\left(\lambda_{0}^{\left(j\right)}\right)}{F_{j}^{\prime }\left(\lambda_{0}^{\left(j\right)}\right)},\;j\in \left\{i,ii,iii\right\}.$$

This approach was prompted by empirical observations that are discussed in Section 5.1 and takes advantage of the monotonicity of $f_{j}\left(\lambda \right)$, which, along with proper choice of the initial value $\lambda_{0}^{\left(j\right)}$, amount essentially to a linearization of the function that yields the optimal threshold around its root.

Noting that the functions $f_{j}\left(\lambda \right)$, j∈{i,ii,iii}, that approximate f(λ) in (21) are all monotonically decreasing, and that they overlap at the edges of the definition intervals for *a* and *b*, that is

(43) $$\begin{array}{ccc} f_{i}\left(\lambda_{a,0}\right) & = & f_{ii}\left(\lambda_{a,0}\right),\;\mathrm{and}\\ f_{ii}\left(\lambda_{b,0}\right) & = & f_{iii}\left(\lambda_{b,0}\right), \end{array}$$

the proposed approach identifies first which approximation case *j* is applicable ({i,ii,oriii}), and determines the one-step numerical solution for the optimal sensing threshold $\lambda_{opt}$ using (42). Once the sensing threshold is found, the 3EED Algorithm 1 is applied with the value $\lambda_{opt}$ to make a decision on the PU activity and channel availability. The approach is formally stated as Algorithm 2.
**Algorithm 2** The 3EED algorithm with adaptive thresholdInput: *N*, α, $\sigma_{n}^{2}$, $\sigma_{s}^{2}$Output: output result1:Use (24) and (25) to obtain $\lambda_{a,0}$ and $\lambda_{b,0}$.2:Use (32) and (33) to obtain $f_{ii}\left(\lambda_{a,0}\right)$ and $f_{ii}\left(\lambda_{b,0}\right)$3:**if**$f_{ii}\left(\lambda_{a,0}\right)<0$ and $f_{ii}\left(\lambda_{b,0}\right)<0$ **then**4:    j=i in (42) and $\lambda_{opt}=\lambda_{opt}^{\left(i\right)}$5:**else if**$f_{ii}\left(\lambda_{a,0}\right)>0$ and $f_{ii}\left(\lambda_{b,0}\right)<0$ **then**6:    j=ii in (42) and $\lambda_{opt}=\lambda_{opt}^{\left(ii\right)}$7:**else if**$f_{ii}\left(\lambda_{a,0}\right)>0$ and $f_{ii}\left(\lambda_{b,0}\right)>0$ **then**8:    j=iii in (42) and $\lambda_{opt}=\lambda_{opt}^{\left(iii\right)}$9:Apply **Algorithm 1** with sensing threshold $\lambda_{opt}$10:**return**$q_{i}$ output by **Algorithm 1**

## 5. Simulations and Numerical Results

In this section, we present numerical results obtained from simulations that support the proposed approach for one-step threshold calculation and illustrate the performance of the 3EED algorithm with an adaptive threshold. The algorithm is also compared to the adaptive CED algorithm in [22] as well as with the “Brute-force” adaptive 3EED algorithm [24] in which an exhaustive search is implemented in Matlab to obtain the value of $\lambda_{opt}$ instead of the proposed one-step approach.

The parameter values used in the simulations are: the number of samples in a sensing slot N=65,537, the SNR γ is between −25 dB and −15 dB, and T=500 slots. The signal transmitted by the PU is implemented using binary phase shift keying (BPSK) modulation, and 2500 sensing slots were considered in each transmission sequence in all simulations.

### 5.1. Sensing Threshold Calculation

**Figure 1:**
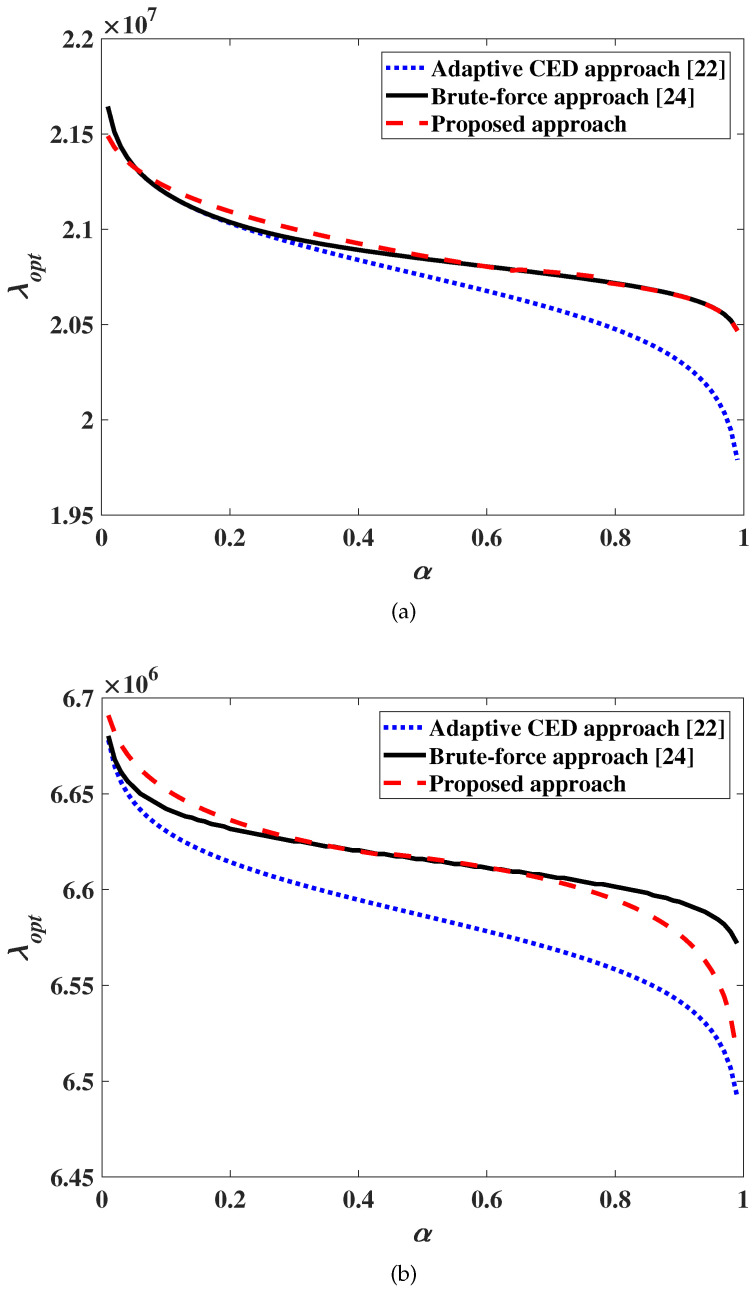
Optimum threshold as a function of α. (**a**) SNR γ=−25 dB, (**b**) SNR γ=−20 dB.

Figure 1 shows the values of the optimal sensing threshold $\lambda_{opt}$ that minimizes the DEP, as a function of α, the spectrum utilization ratio, for SNR values of −25 and −20 dB, corresponding to the proposed approach discussed in Section 4, the “Brute-force” approach in [24], and the adaptive CED approach in [22]. From this figure we note that the values of the sensing threshold obtained using the proposed approach closely match those obtained using the “brute-force” approach [24] for values of α between 0.2 and 0.7, in particular for α around 0.5, and support the application of the numerical approach presented in Section 4 for threshold calculation with minimal overhead. We also note that, according to studies performed on GSM channels [30] or in the industrial, scientific and medical (ISM) bands [31], this is the range of values for the spectrum utilization ratio α that is of practical interest for SU access to licensed spectrum.

### 5.2. Decision Error Probability

**Figure 2:**
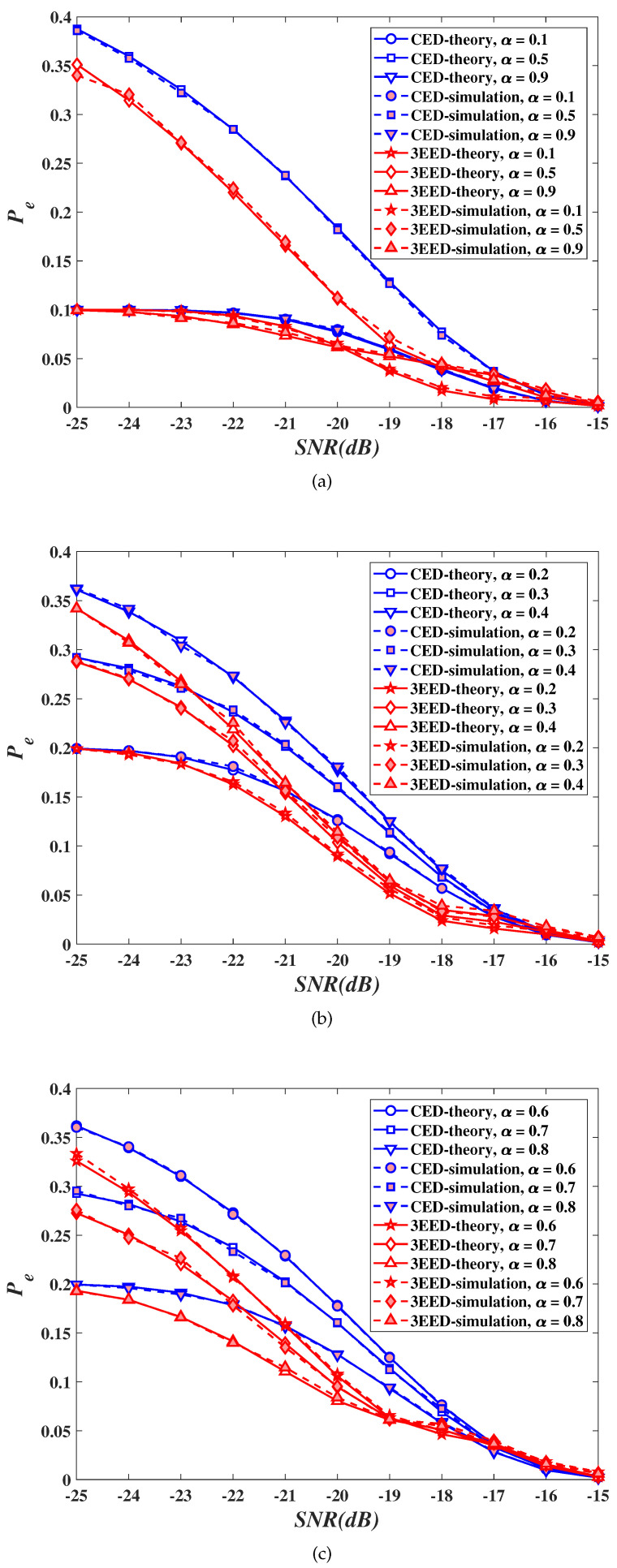
Decision error probability (DEP) for adaptive algorithms as a function of signal-to-noise ratio (SNR). (**a**) α=0.1,0.5, and 0.9; (**b**) α=0.2,0.3, and 0.4; (**c**) α=0.6,0.7, and 0.8.

Figure 2 shows the values of the DEP $P_{e}$ as a function of the SNR γ for different values of the spectrum utilization ratio α, for the proposed 3EED algorithm with an adaptive threshold as well as for the adaptive CED algorithm in [22]. Based on the plots shown in Figure 2 we first note that the values of the DEP obtained through Monte Carlo simulations closely match the analytical values of the DEP implied by (6) for the proposed 3EED algorithm with an adaptive threshold or by the corresponding DEP expression in [22], for all SNR and α values. We also note that the proposed 3EED algorithm with an adaptive threshold outperforms the adaptive CED algorithm in cases of practical interest corresponding to spectrum utilization ratios above 0.2 and below 0.7, with maximum gain of the proposed algorithm in terms of the DEP of about 1 dB achieved for α around 0.5. Furthermore, for both the proposed 3EED algorithm with an adaptive threshold and the adaptive CED algorithm, the value of the DEP becomes less sensitive to changes in the spectrum utilization ratio α as the SNR values increase.

**Figure 3:**
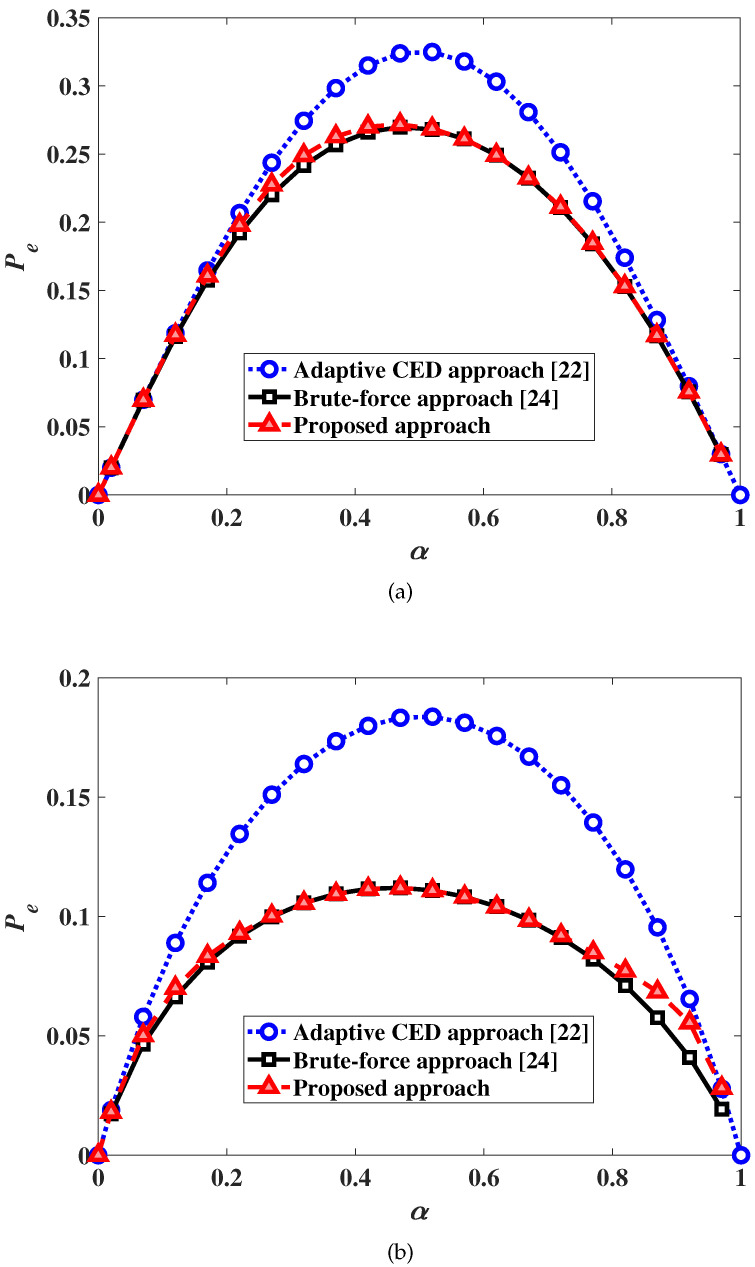
DEP as a function of the spectrum utilization ratio α. (**a**) SNR γ=−23 dB; (**b**) SNR γ=−20 dB.

Figure 3 shows the dependence of the DEP on the spectrum utilization ratio α for two different SNR values γ=−23 dB and −20 dB. As it can be observed, the DEP decreases with increasing SNR for both the proposed 3EED algorithm with an adaptive threshold and the adaptive CED algorithm. Furthermore, the proposed algorithm outperforms the adaptive CED one for all values of the spectrum utilization ratio α. We note that, for each value of α, the corresponding DEP values are minimum since the optimal sensing threshold is used for that α, as discussed in Section 3.

## 6. Conclusions

A new ED algorithm for spectrum sensing in CR systems is presented in the paper. The proposed algorithm employs an adaptive sensing threshold that is optimized to minimize the DEP and has minimal overhead, since the value of the optimal threshold is found through a one-step iterative method.

Numerical results obtained from simulations are presented to illustrate the performance of the proposed algorithm and to compare it to the adaptive CED algorithm. The results show that the proposed algorithm outperforms the CED algorithm for spectrum sensing, resulting in lower values for the DEP for all values of the spectral utilization ratio that are of practical interest for CR systems providing SU access to licensed spectrum.

We intend to implement and validate the proposed algorithm using SDR platforms from the USRP family. In this context, we will also focus on the optimization of the expressions used in the algorithm to estimate the decision threshold for minimizing the computational complexity and the sensing time.
